# How to *Stop* Cognitive Processes is as Important as How to *Start* Them. Commentary on Dipoppa et al.

**DOI:** 10.5709/acp-0200-y

**Published:** 2016-12-31

**Authors:** Matthias H. J. Munk

**Affiliations:** 1 Department of Psychiatry, Eberhard-Karls-Universität, Tübingen, Germany; 2 Systems Neurophysiology, Technische Universität, Darmstadt, Germany

**Keywords:** working memory, active storage neuronal implementation, oscillations, cognitive process

## Abstract

We are used to dealing with concepts which provide explanations for how cognitive processes are initiated. But we hardly ever spend time on trying to explain how such processes are turned off again and, thus, do not compromise subsequent processes.

## COMMENTARY

 In their article, Dipoppa, Szwed, and Gutkin (2016) integrated an impressively large body of results from studies on neuronal mechanisms of working memory (WM) in both human and nonhuman primates. Most importantly, they carefully consider, from a systems neuroscience approach how several neuronal processes need to co-operate in order to provide a flexible and, at the same time, reliable mechanism to temporarily maintain information in brain circuits. After distinguishing various definitions of WM, they explain *active storage* (WMAS), as defined in line with Cowan (2008) as temporarily activated long-term memory. Given the active nature of memory maintenance, its content can be modified by cognitive processes, which is usually dubbed ”manipulation”. Therefore, in addition to the classical core operations of WM >*LOAD*<, >*MAINTAIN*<, and >*READ-OUT*<, the following operations are additionally required: >*PREVENT*< distractors, >*RESTRAIN*<, and active deletion, dubbed >*CLEAR*<. The authors not only review a lot of literature providing experimental evidence for putative neuronal implementations of these additional operations but also test them explicitly in their model (Dipoppa & Gutkin, 2013), which provides evidence for two different WM tasks being served by a unified gating mechanism. 

 What is really new here is the authors’ approach to the role of oscillations. Although it has often been mentioned since the first observations of oscillations in the brain, and in particular in the cortex, the authors convincingly show that oscillations are instrumental for organizing processes and do not constitute an alternative mechanism for coding (McLelland & VanRullen, 2016) or maintaining information (Lundqvist et al., 2016). Since their discovery by Hans Berger in the human brain (1929), oscillations are known to reflect states of the brain. The *alpha rhythm* has been described as the brain’s signature when the subject under study closes the eyes. In modern terms, alpha occurs when the processing power of the visual cortex is not needed and disappears immediately when fresh sensory input reaches the cortex upon opening the eyes, giving way to faster oscillations. Likewise, fast oscillations, for example, in the gamma-frequency range, have been shown to be state-dependent (Herculano-Houzel, Munk, Neuenschwander, & Singer, 1999). It is more controversial whether fast oscillations are instrumental for information coding (Lundqvist et al., 2016) or, as suggested by Dipoppa et al. ( 2016), oscillations serve the organization of memory processes. However, the cited evidence is altogether correlative, meaning that instead of information coding, gamma oscillations may mediate attentional bias (Wang, 2010) or may actually be required for driving plasticity (Harris, Csicsvari, Hirase, Dragoi, & Buzsaki, 2003 ; Traub et al., 2005), so that after cessation of sustained activity, hidden layers have saved information so that new sensory activation enables the prefrontal network to recall stored information (Stokes, 2015). 

 In the article of Dipoppa et al. (2016), oscillations are considered as organizing elements which set the memory supporting brain circuits into a mode by which they can coordinate the different neuronal processes required for the sequence of memory operations (see above). The most intuitive function of oscillations appears to be *gating*, which is considered as a selection process in which the periodic modulation of neuronal activity serves as a temporal filter. This way, signals can be either sent on into storage or be suppressed by either not reaching threshold for attention or being channeled through to executive areas. It has been shown that oscillations in the alpha-frequency range can exert a strong inhibitory effect on signal transmission and processing (Sauseng et al., 2009), though it is not entirely clear how this happens. Inhibitory gating by alpha oscillations may occur because of the induction of a slower than usual rhythm predominating during active processing. This may simply stop the faster one, for example, a gamma rhythm, which is known to support spatial integration across large dendritic trees (Zador, Agmon-Snir, & Segev, 1995) and thus facilitates spike output. 

 Another completely independent observation of oscillations in the alpha-frequency range concerns the frontal cortex during loss of consciousness: When anesthesia is induced by propofol, a strong, highly synchronized 10-Hz rhythm occurs across the frontal and prefrontal cortex which immediately ceases when anesthesia wears off ( Purdon et al., 2013). Of course, there is no simple relation to the observation of slow rhythms supporting the interruption or stopping of executive functions, but this finding can be considered as another puzzle piece in understanding fast cessation of integrative function, even though it is pharmacologically induced. Slow oscillations in the delta- and theta-frequency range have been shown to mediate cross-modal attention which includes the amplification of relevant and the suppression of irrelevant inputs (Schroeder & Lakatos, 2009). More recently, the interruption and/or cessation of WM processes have been studied more intensively to unravel the mechanisms by which, for example, surprise suppresses ongoing motor and cognitive processes. Wessel et al. (2016) investigated the mechanisms by which surprise interrupts auditory WM by a short epoch of unique birdsong instead of a standard sine-wave tone. Intriguingly, this study provided extra- and intracranial recordings to show fronto-central slowing of the EEG, in the form of delta waves, which occurred during incorrect trials. Single-trial analyses revealed that this low-frequency activity mediated the decremental effect of surprise on WM accuracy. 

 Taken together, numerous studies have finally begun to investigate the successful discontinuation of neuronal processes underlying cognitive functions, such as attention and working memory. Stopping is particularly important in the case of WM to achieve the necessary flexibility of processes supporting information storage. Apart from their broad scope, the exciting aspect of the review by Dipoppa et al. (2016) is the integration of multiple operations in a reconstructive model of WM which shows that the proposed mechanisms for individual operations fit together and provide a setting for investigating details of the implementation, which cannot easily be studied in their in vivo implementation. Thus, modeling does not only allow for reconstructing our new knowledge, but may also provide important cues for guiding new experiments. 
